# Development and Characterization of a New TILLING Population of Common Bread Wheat (*Triticum aestivum* L.)

**DOI:** 10.1371/journal.pone.0041570

**Published:** 2012-07-23

**Authors:** Liang Chen, Linzhou Huang, Donghong Min, Andy Phillips, Shiqiang Wang, Pippa J. Madgwick, Martin A. J. Parry, Yin-Gang Hu

**Affiliations:** 1 State Key Laboratory of Crop Stress Biology in Arid Areas and College of Agronomy, Northwest Agricultural and Forestry University, Yangling, Shaanxi, China; 2 Centre for Crop Genetic Improvement, Department of Plant Science, Rothamsted Research, Harpenden, Herts, United Kingdom; 3 Institute of Water Saving Agriculture in Arid Regions of China, Northwest Agricultural and Forestry University, Yangling, Shaanxi, China; Umeå Plant Science Centre, Sweden

## Abstract

Mutagenesis is an important tool in crop improvement. However, the hexaploid genome of wheat (*Triticum aestivum* L.) presents problems in identifying desirable genetic changes based on phenotypic screening due to gene redundancy. TILLING (Targeting Induced Local Lesions IN Genomes), a powerful reverse genetic strategy that allows the detection of induced point mutations in individuals of the mutagenized populations, can address the major challenge of linking sequence information to the biological function of genes and can also identify novel variation for crop breeding. Wheat is especially well-suited for TILLING due to the high mutation densities tolerated by polyploids. However, only a few wheat TILLING populations are currently available in the world, which is far from satisfying the requirement of researchers and breeders in different growing environments. In addition, current TILLING screening protocols require costly fluorescence detection systems, limiting their use, especially in developing countries. We developed a new TILLING resource comprising 2610 M_2_ mutants in a common wheat cultivar ‘Jinmai 47’. Numerous phenotypes with altered morphological and agronomic traits were observed from the M_2_ and M_3_ lines in the field. To simplify the procedure and decrease costs, we use unlabeled primers and either non-denaturing polyacrylamide gels or agarose gels for mutation detection. The value of this new resource was tested using PCR with RAPD and Intron-spliced junction (ISJ) primers, and also TILLING in three selected candidate genes, in 300 and 512 mutant lines, revealing high mutation densities of 1/34 kb by RAPD/ISJ analysis and 1/47 kb by TILLING. In total, 31 novel alleles were identified in the 3 targeted genes and confirmed by sequencing. The results indicate that this mutant population represents a useful resource for the wheat research community. We hope that the use of this reverse genetics resource will provide novel allelic diversity for wheat improvement and functional genomics.

## Introduction

Wheat is an important food crop world-wide. However, many traits that are important for wheat production would benefit from the ability to understand and modify the function of specific genes [1], [2]. Recently, genome sequencing programs for many plant species [3]–[5] has led to the availability of a large number of genomic sequences in public databases which subsequently has encouraged the development of reverse genetics tools [6]. Reverse genetic approaches use genomic sequence information to identify sequence variations in genes of interest and then analyze the phenotypic effects conferred by the mutant alleles to determine the function of a gene. Several reverse genetic tools are currently used for this purpose, such as T-DNA or transposon insertion, which have greatly assisted functional genomics in model species [7]–[10]. Unfortunately, these resources are still not available in wheat [11], [12]. Additionally, most mutations resulting from these insertional methods are likely to be knockout mutants rather than allelic series of mutants with partial loss of function, and thus will not produce the range of mutation strengths necessary for crop improvement [11]. RNAi has emerged as an effective gene knockout/knockdown tool for many plants and is also a useful technique in wheat [12], [13]. However, RNAi is not wholly reliable in generating stable reductions in target gene suppression. Furthermore, both insertion mutagenesis and RNAi require genetic transformation which is limited to a few varieties in wheat and still has a lack of acceptance by consumers in many countries [2], [11], [14], [15]. Thus, TILLING (Targeting Induced Local Lesions IN Genomes), which combines chemical mutagenesis with high-throughput genome-wide screening for point mutations in genes of interest, has been developed in response. This methodology may be preferable to other reverse genetics approaches for various reasons. EMS (ethyl methanesulfonate) produces a large spectrum of mutations, including truncations and missense mutations, allowing more flexibility than insertional mutagenesis or transgenesis [16], [17]. Furthermore, EMS can create random point mutations at high density in polyploid plants. This allows multiple alleles of a specific gene to be obtained in a small population regardless of the genome size [18]–[21].

The TILLING method is useful for both functional genomics and crop improvement. Although the DNA sequence of a gene may provide enough information to infer function, such predictions must be validated phenotypically [22]. TILLING can provide the practical verification needed for this type of sequence-driven hypothesis [16]. The importance of this locus-to-phenotype method is demonstrated by the success of the Arabidopsis TILLING Project, a high-throughput service platform for gene functional analysis [17], [22]. TILLING also finds application in crop improvement, as the mutants identified by TILLING can be readily utilized in conventional breeding programs since it is non-transgenic and the novel variations can be inherited stably [2], [12], [16], [23]. TILLING resources have been developed for several crop species such as maize [23], barley [24]–[26], rice [19], [27], sorghum [28], soybean [29], potato [30], peanut [31], *brassica napus* [32], *brassica rapa* [33] and tomato [6]. In wheat, TILLING has been applied to both tetraploid and hexaploid varieties and is used to create a near-null waxy phenotype by targeting the *waxy* genes [15]. By intercrossing two truncation mutants of the *waxy* homoeologs identified through TILLING, a complete waxy phenotype was produced [2]. Moreover, the polyploid nature of wheat makes it requires a relatively small population for TILLING analysis, which further improves the cost-efficiency for maintaining the population and screening the desired mutations [2], [11], [12].

However, most detection systems for TILLING rely on the use of high-throughput electrophoresis equipment, such as the LI-COR DNA analyzer or ABI genetic analyzer, which use fluorescent end-labeled primers and are relatively expensive for individual laboratories [2], [12], [15], [16], [34], [35]. These requirements could be barriers to the adoption of this approach. We sought to simplify the detection method to make the technique more accessible to researchers in developing countries where access to those instruments could be limiting. Recently, alternative, inexpensive detection systems for TILLING have been tested using agarose gel and non-denaturing polyacrylamide gel (staining with ethidium bromide) [12], [36], and have been used to detect EMS-induced mutations in large libraries [2], [12].

Practically, the capability to investigate gene function and to use novel mutation for wheat improvement will become increasingly crucial as more wheat sequence information is available. In addition, genetic resources developed in different genetic backgrounds and with different EMS concentrations can increase the chance of obtaining a broad range of alleles. Thus, a diverse set of publicly available wheat mutants is needed to allow more efficient validation and application of candidate genes [12] and increase the possibility of identifying a greater number of mutations of interest. Here we report the construction of a new EMS-induced TILLING population comprising 2,610 M_2_ individuals of the common wheat cultivar ‘Jinmai47’. Phenotypes of mutant lines in the population were characterized and the average mutation frequency for this population was determined by PCR with RAPD and ISJ primers, and TILLING of three different gene fragments, respectively. We modified and compared the three non-fluorescence detection methods (agarose gel, non-denaturing polyacrylamide gel stained with ethidium bromide and with silver solution) for finding an optimum strategy of TILLING screenings for researchers under similar laboratory conditions to us. It is suggested that the new TILLING population generated from wheat cultivar ‘Jinmai47’ is a resource with potential for use in fundamental research as well as for applied breeding.

## Results

### Establishment of the TILLING Population

To determine a suitable EMS concentration for mutagenesis, we conducted germination tests following treatment with EMS for 18 h at concentrations of 0.8, 1.0 and 1.2% (v/v) according to previous experience in different wheat cultivars [2], [11], [12], [15]. We determined that the germination rates of 25% and 8% following 1.0% and 1.2% treatments were too low. With a 0.8% EMS treatment, the germination rate was about 40%, and 1350 M_1_ plants were grown to maturity, harvested and advanced to the M_2_ generation. We extracted genomic DNA from two or three single plants of M_2_ lines separately. In a preliminary experiment, the genomic DNA from five to ten individuals was pooled for mutation detection, but did not give clear digested bands by PAGE or agarose gel electrophoresis (data not shown). Therefore, in subsequent experiments samples were pooled fourfold, which gave favorable results. Finally, DNAs from a total of 2610 M_2_ plants were pooled in groups of four for convenient screening.

### Phenotyping of the mutagenized population

In total, 2610 M_2_ individuals were scored for their phenotype. All lines were observed from the seedling stage to full maturity with specific emphases on altered seedling phenotypes, plant height, spike morphology, fertility, heading date, and other obvious variations from the wild-type ‘Jinmai47’. About 4% of the surveyed M_2_ lines displayed noticeable phenotypes (distinctive from Jinmai47), which are listed in Table 1.

**Table 1:** Frequency of typical mutations observed among the 2,610 M_2_ individuals screened.

Phenotype description	Number of mutants observed	Frequency
dwarf and semi-dwarf	35	1.34%
spike morphology	16	0.61%
tiny plants	8	0.31%
albinism	7	0.27%
late heading	7	0.27%
lower fertility	5	0.19%
few tillers	4	0.15%
seedling lethal	4	0.15%
erect leaf	3	0.11%
wide leaf	3	0.11%
seed size	3	0.11%
deep green leaf	2	0.08%
disease sensitive	2	0.08%
early senescence	2	0.08%
Single tiller	2	0.08%
narrow leaf	1	0.04%
yellow green leaf	1	0.04%
wax leaf	1	0.04%
multiple tillers	1	0.04%
coleoptiles shape	1	0.04%

**Figure 1:**
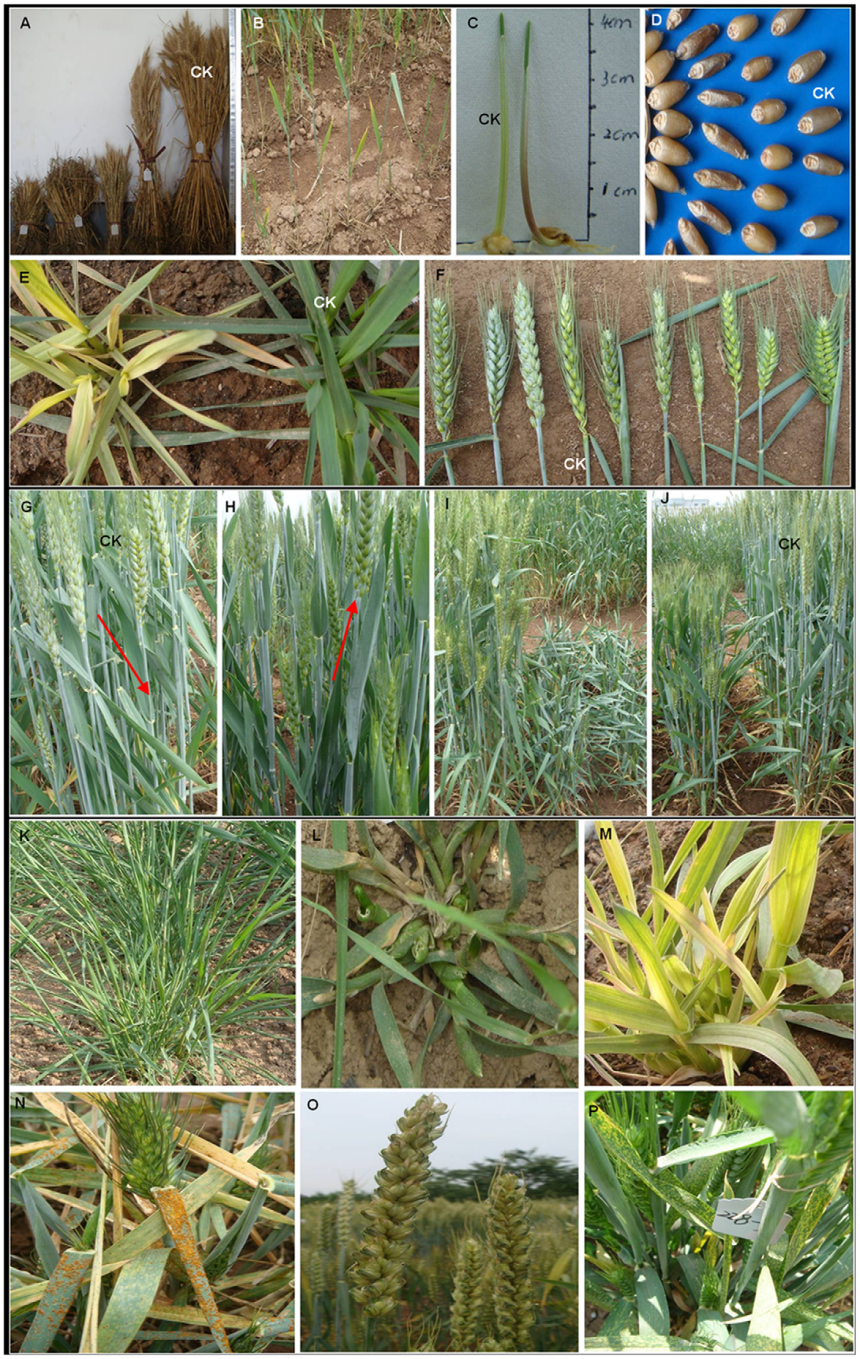
Mutant phenotypes observed in the M_2_ and M_3_ wheat plants. Mutant phenotypes: (a), (i), (j) dwarf and semi-dwarf; (b) single tiller; (c) coleoptile color; (d) seed size; (e), (m) albinism; (f) spike morphology; (g)(h) erect leaf; (k) narrow leaf; (l) strange leaf morphology; (n) disease sensitive; (o) large spikes with short awns; (p) yellow spots on leaves.

Variants in plant height comprised 32% of all visible phenotypes noted and mainly yielded different kinds of dwarfism. Spike morphology diversity comprised the second most frequent type of mutation. Tiny plants, which are different from dwarf plants in having reduced size of all organs,accounting for 7% of all the phenotypic mutants, and these mutants were always associated with a delayed heading date and a lower fertility. Among the plants classified as leaf color mutants, more than half exhibited albinism, yellow-green or deep-green leaves. In addition to these frequent mutation traits, a number of other mutation phenotypes such as heading date, number of tillers, disease resistance, early senescence, seed size, and leaf shape were also observed. A selection of the mutant phenotypes observed in the field is presented in Figure 1. The frequency and the wide range of the phenotypic mutations observed in this population implied that it would be a valuable resource for screening desired wheat mutants needed in forward and reverse genetic researches.

### Estimation of the mutation frequency by PCR

The number of visible phenotypes in a mutant population can indirectly reflect the number of mutations in the genome. However, it is not realistic to estimate mutation rate in this way as it is affected by gene redundancy, synonymous mutations, mutations in non-coding regions and the fact that wheat is hexaploid. Therefore, RAPD (Random Amplification of Polymorphic DNA) and ISJ-PCR method was used for better approximating the mutation frequency.

**Figure 2:**
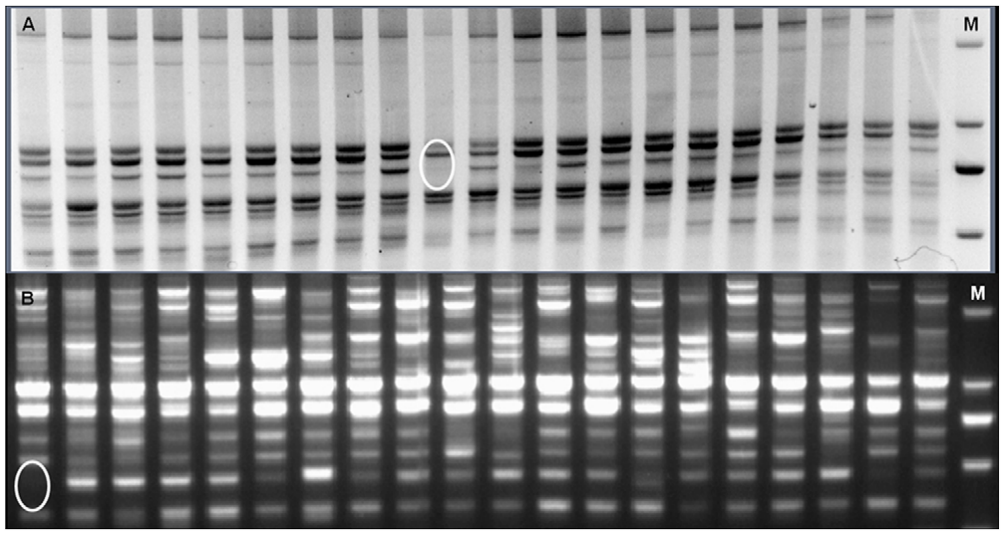
An example of RAPD banding pattern obtained by primer R1 and primer IT31. W: wild type; m: mutagenised lines; M: molecular weight standards DL2000.

The PCR reactions were standardized with genomic DNA of wild type Jinmai47 as the template for three short random primers and three intron splice junction (ISJ) primers. The variations among 300 independent M_2_ lines with the wild type were detected using the corresponding PCR conditions. The bands that differed from the wild type (non-mutagenized Jinmai 47) were recorded (Fig. 2) and the mutation frequency was calculated (Table 2). In general, the mean mutation frequency was estimated to be approximately one mutation per 34 kb.

**Table 2:** Primer sequences of the 6 primers used and the mutation frequency detected.

Primer	Sequence (5′- 3′)	Primer length	No. of bands in wild type	No. of mutation bands	M_2_ plants screened	Mutation frequency
A-09	GGGTAACGCC	10	6	1	300	1/36 kb
A-10	GTGATCGCAG	10	8	3	300	1/16 kb
UBC3	CCTGGGCTTA	10	7	4	300	1/10 kb
R1	TCGTGGCTGACTTCACTG	18	8	1	300	1/86 kb
E4	GAATTCCAGCCTGCA	15	7	2	300	1/31 kb
IT31	GAAGCCGCAGGTAAG	15	6	2	300	1/27 kb

### Wheat TILLING platform

**Figure 3:**
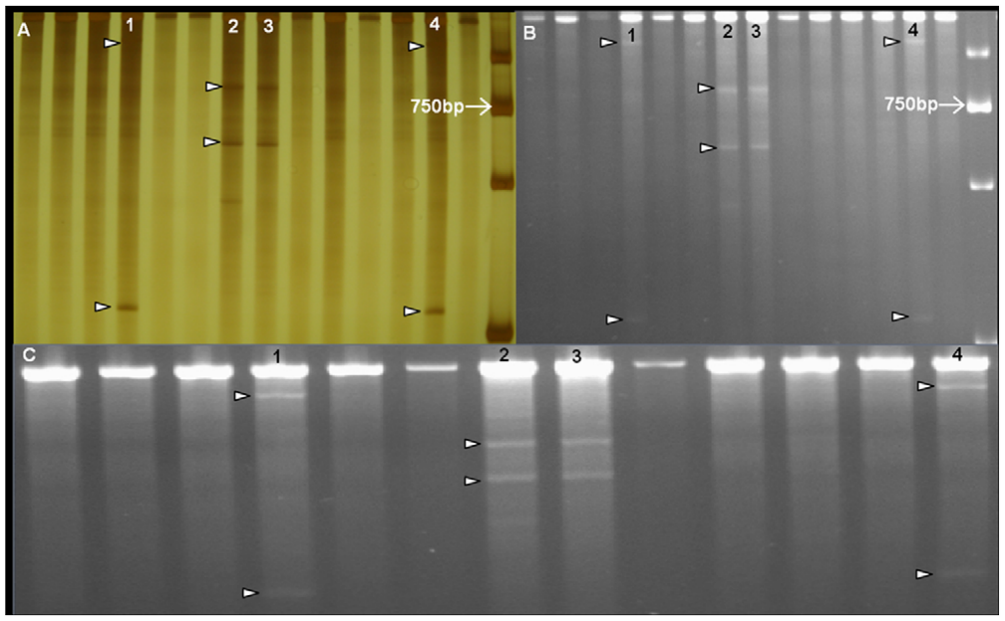
Digested bands detected with non-denaturing polyacrylamide gels stained with either silver (a) or ethidium bromide (b) and agarose gels stained with ethidium bromide (c). Putative mutations in the pools (1, 2, 3, 4) are identified by the presence of two bands (indicated by white arrows), with sizes adding up to the full length PCR product. (a). Non-denaturing polyacrylamide gel stained with silver; (b). Non-denaturing polyacrylamide gel stained with ethidium bromide; (c). Agarose gels stained with ethidium bromide.

To develop a fast and cost-effective method for mutation detection, mismatch-specific nuclease digestion of heteroduplex DNA followed by non-denaturing polyacrylamide gels stained with either silver or ethidium bromide and agarose gels stained with ethidium bromide were tested. A 1496 bp region of the *Ppd-D1* gene was screened in 512 M_2_ plants using the three methods and the results were compared. It was found that the non-denaturing polyacrylamide gels stained with silver could most efficiently detect the bands produced by CJE (Celery Juice Extract) [34], [37] digestion of the re-annealed PCR products from the fourfold DNA pools, while that stained by ethidium bromide resulted in a little lower resolution, and the agarose gels stained by ethidium bromide also could obtain similar results (Fig. 3). However, the detection effects of non-denaturing polyacrylamide gels with silver staining were more favorable for visualizing the smaller bands generated with a little higher sensitivity than that of agarose gels stained by ethidium bromide. In addition, the non-denaturing polyacrylamide gels by silver-staining allowed the distinction of digested bands in an eightfold pool, making it comparable to the LI-COR system (data not shown).

Thus, the fast and efficient screening method based on the non-fluorescence system including polyacrylamide gels and agarose gels was developed. These gels take less time from preparation to finish a gel than the LI-COR system (about 5 h), and also have the advantages of easy operation, low cost and high throughput. They were demonstrated to be convenient and effective in mutation detection.

### Mutation detection with three candidate genes

Screening of the targets of three candidate genes of *Ppd-D1*, *Rubisco activase A* and *Rubisco activase B* in 512 random plants from the M_2_ TILLING population obtained 15, 7, and 9 mutations, respectively. Sequencing confirmed that all of the mutations were G to A or C to T transitions as expected from alkylation by EMS [18], [23], [33]. This translated into an average mutation frequency of at least one mutation per 47 kb (Table 3).

**Table 3:** Mutation detection and estimation of mutation frequency in three candidate genes of *Ppd-D1*, *Rubisco activase A* and *Rubisco activase B* by TILLING analysis.

Gene	Amplicon Size(bp)	M_2_ plants screened	Mutation					Frequency*(kb)
			Total	Intron	Silent	Missense	Truncation	
*Ppd-D1*	1496	512	15^a^	9	1	5	-	1/44
	1496	512	11^b^	7	1	3	-	1/60
	1496	512	14^c^	8	1	5	-	1/47
*RubiscoA*	946	512	7^a^^,^ c	2	3	2	-	1/54
*RubiscoB*	959	512	9^c^	4	2	3	-	1/43

a , mutation detected by non-denaturing polyacrylamide gels stained with silver;

b , mutation detected by non-denaturing polyacrylamide gels stained with ethidium bromide;

c , mutation detected by agarose gels stained with ethidium bromide.

* For calculation of the mutation frequency, 100 bp sequences from each end were removed due to the base ambiguity.

### Characterization of the identified *Ppd-D1* mutations

**Figure 4:**
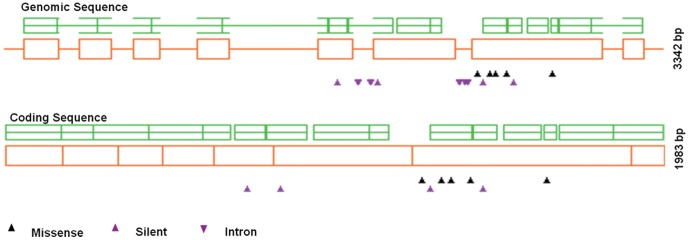
Type and distribution of induced mutations discovered in *Ppd-D1* amplicon.

In the *Ppd-D1* mutants, nine C to T and six G to A transitions were found. Four of these created silent mutations, but six led to amino acid changes (Fig. 4). These mutations were evaluated by SIFT (Sorting Intolerant From Tolerant; http://blocks.fhcrc.org/sift/SIFT) and PSSM (Position Specific Scoring Matrix; http://www.proweb.org/parsesnp/) scores (Table 4).

**Table 4:** Analysis of mutations identified in the *Ppd-D1* gene.

Line	Nucleotide change	Amino acid change	PSSM	SIFT	Zygosity*	Type
P401-2	C1687T	S242S	-		Het	
P346-1	C1793T	Intron	-	-	Het	
P20-1	C1856T	Intron	-	-	Het	repeat
P20-2	C1856T	Intron	-	-	Hom	repeat
P302-1	G1895A	L276L	-	-	Hom	
P401-1	G2309A	Intron	-	-	Het	
P144-1	G2337A	Intron	-	-	Het	
P283-1	C2353T	Intron	-	-	Hom	
P127-1	G2401A	G418S	-	0.63	Het	repeat
P127-2	G2401A	G418S	-	0.63	Het	repeat
P186-1	C2427T	C426C	-	-	Hom	
P283-1	C2462T	T438M	12.0*	0.01*	Hom	
P229-1	C2489T	S447F	16.9*	0.00*	Hom	
P271-1	C2548T	P467S	14.4*	0.28	Het	
P192-1	C2586T	G479G	-	-	Het	
P190-1	G2777A	G543E	−0.7	1.00	Hom	repeat
P190-3	G2777A	G543E	−0.7	1.00	Hom	repeat
P71-1	G2779A	G544S	−0.4	1.00	Het	

* Het, heterozygote; Hom, homozygote.

PSSM or SIFT scores of mutation lines with star (*) are predicted to be damaging to protein function. Mutation with PSSM score larger than 10 indicates that the mutation is more likely to have a damaging effect on the protein function. Mutation with SIFT score less than 0.05 is predicted to be deleterious.

The mutations in line p283-1 is predicted to be less damaging to protein function than the mutation in p229-1 by PSSM and SIFT scores, both lines (homozygous *Ppd-D1* mutant lines) displayed a similarly delayed late heading and flowering date. Spike development observation by microscopy showed that the late reproductive phase of stem elongation (initiation of the terminal spikelet to anthesis) of the two lines had been lengthened when compared to the wild-type or to sibling mutation lines under the same photoperiod condition. We examined the effects of the mutation in the F_2_ population from p283-1×wild type Jinmai47. The data showed that the non-mutant F_2_ segregants and wild type plants have a similar duration of spike development, while the homozygous mutant F_2_ segregants exhibited a significant delay in flowering time. However, the homozygous mutant F_2_ plant did not have an optimal duration of the reproductive development phase (the anthesis date was too late) and was not satisfactory for our breeding purpose.

Repeated mutations were identified in the *Ppd-D1* gene within sibling M_2_ lines (Table 4) as C1856T, G2401A and G2777A presented in different individuals of M_2_ lines of p20, p127 and p190, respectively. Additionally, two individuals (p190-1 and p190-3) of the M_2_ line p190 had the same two missense mutations (G2777A, G543E), and another individual (p190-2) had the same genomic sequence with wild type, this is more likely the result of genetic segregation. However, different mutations were also found in the sister individuals of the same M_2_ lines. One individual (p401-2) of the line p401 had the silent mutation C1687T, whereas another individual (p401-1) had the mutation G2309A in the intron region. Although this happened once only in the TILLING screening, it implied that these individuals might originate from different meristem cells. These results illustrated the chimeric nature of mutagenized M_1_ plants, resulting in M_2_ individuals from the same M_1_ plant potentially having different mutations. Although this was quite rare, it suggested that screening of multiple individuals derived from the same M_1_ plant might find some new mutations. Furthermore, two different mutations were identified in the individual p283-1 with one in an intron region and the other causing an amino acid change, indicating a high mutation density of the target.

Although we did not find any truncation (nonsense or splice junction) mutation in the targets, many non-synonymous mutations were revealed, highlighting the power of this detection approach and the TILLING population for functional gene analysis.

## Discussion

The TILLING approach is a non-transgenic method for functional genomic studies and crop improvement, based on mutagenesis followed by sensitive molecular screening. Here, a TILLING population of Jinmai 47 has been developed in wheat using the chemical mutagen, EMS. To obtain as many mutations as possible in the M_2_ lines, we chose two or three individuals per M_2_ line for developing the Tilling population. This is different from other wheat Tilling programs that only used one seed from each M_1_ plant to generate M_2_ plants [11], [12]. The latter approach will lose some mutations due to segregation in the M_2_ lines, and screening of multiple individuals per M_2_ line can therefore identify additional mutations [2].

Characterization of this population through PCR with RAPD and ISJ markers and by TILLING screening of the targets of three candidate genes revealed the mutation densities of at least one mutation per 34 kb and 47 kb, respectively. These mutation densities are lower than those found by Slade et al. (1/∼24 kb) [15], and Dong et al. (1/∼30 kb) [2], but similar to that reported by Uauy et al. (1/∼49.4 kb) [12] and Parry et al. (1/∼49 kb) [11] in wheat using similar EMS concentrations. There are many factors that may affect the induced mutation frequency, such as mutagen concentration, exposure time, environmental effects and the genotype of the target species [28]. A concentration of 1.2% EMS was used in our pilot TILLING study in attempting a high mutation frequency, but only ∼8% of the M_1_ seeds germinated under the treatment. EMS is reported to have a strong preference for 5′-PuG-3′sites, and for a middle G base within a stretch of three or more G bases [28], [38]. Therefore, the difference observed in these studies is likely dependent on the slight differences in EMS doses, different GC content of the target regions and also the different genotypes employed in these studies. Additionally, the mutation frequency may be changed after additional screening of different genes or evaluation of more mutant lines from the population. Changing the detection method (such as using the LI-COR or ABI3700 systems) and the pooling strategy used may also affect the observed mutation frequency.

Wheat is especially well suited for TILLING because it tolerates a much higher mutation density than diploid plants due to its polyploid nature [12]. These results and other wheat TILLING studies [2], [11], [15] indicate that wheat tolerates very high mutation frequencies (1 in 20∼50 kb). Compared with the mutation frequency of EMS mutagenesis in Arabidopsis (1/170 kb) [18], sorghum (1/526 kb) [28], barley (1/500 kb) [26], sunflower (1/475 kb) [21], tomato (1/574 kb) [6], [39], melon (1/573) [40] and rice (1/300∼600 kb) [19], this indicates that a TILLING population of hexaploid wheat, where variations in all genes could be found with a high redundancy, could be produced with a relatively small population size, which requires much less advanced logistics in handling and maintenance. This situation is also observed in a TILLING population of hexaploid oat [41].

The redundant genome constitution of wheat could be a potentially complex problem when identifying specific recessive phenotypes from the TILLING population. The phenotype of a single mutant may be masked by the wild-type homoeologue(s) present in the other two genomes. However, it should be noted that the three genomes often contribute differentially to the expression of certain genes [42], therefore recessive phenotypes from single mutations is possible. Thus, about 3.8% of the M_2_ lines that we developed demonstrated altered morphologies in comparison with the wild-type (Figure 1). Some of the mutant lines (45%) showed a single altered trait, while more than half of the lines displayed multiple mutant traits (always with variant plant size). This is much higher than that described by Slade et al. [15] with only about 0.5% abnormal phenotypes observed and similar to that of 5% reported in the TILLING population of hexaploid oat [41], but lower than that reported in barley (20%) [26] or tomato (39%) [6]. This all indicate that the polyploid plants have higher mutation density, but have lower phenotype frequency due to the compensation effect among the genomes while diploid plants respond in a nearly opposite manner (Table 5). This conclusion may provide more information for producing new TILLING resources of plants. However, mutants with variations in multiple traits were counted only once in this study according to the major mutation trait. This leads to an underestimation of the mutation frequency at the phenotypic level. Moreover, some phenotypic traits, like root or seed texture, were not taken into account; therefore the phenotypic mutation frequency detected in the M2 generation may have been lower than the true frequency of detectable phenotypes.

**Table 5:** The mutation density and germination rate in published TILLING populations of different species under different EMS dosage treatments.

Species	Ploidy	Concentration of EMS (%)	Mutation density(kb)	Frequency of Phenotypic variation in M2 (%)	Germination rate (%)	Reference
Wheat	6×	0.75; 1.0	1/24	0.5	-	[15]
	4×	0.75	1/40	0.5	-	[15]
Wheat	6×	0.9∼1.0	1/38	-	50∼60	[12]
	4×	0.7∼0.75	1/51	-	50∼60	[12]
Wheat	6×	0.5∼0.7	1/37∼1/23	-	60∼80	[2]
Wheat	6×	0.8	1/34; 1/47	3.8	40	Author's data
Wheat	2×	0.24	1/1300	>20	51	[22]
Oat	6×	0.9	1/40∼1/20	5	37	[39]
Peanut	4×	0.4; 1.2	1/931; 1/1067	-	30∼50	[31]
Rice	2×	0.8/1.0; 1.6	1/2000; 1/1000	8.3	-	[27]
Sorghum	2×	0.25	1/526	>10*	normal	[28]
Tomato	2×	0.7; 1.0	1/574; 1/322	39	80; 51	[6]
Barley	2×	0.2∼0.3	1/1000	20*	70∼50	[24]
Barley	2×	0.2∼0.63	1/500	20	92∼40.5	[26]
Sunflower	2×	0.7	1/475	4.79	65	[21]
Brassica	2×	0.3; 0.4	1/56; 1/67	-	83; 60	[33]
Brassica	4×	0.3; 0.6	1/130; 1/41	12.6; 23.2	95; 82.3	[32]
Arabidopsis	2×	0.2∼0.4	1/300	-	-	[18]

* in M_3_ plant.

To evaluate the mutation density more directly on the DNA level in the whole genome, a modified RAPD method with ISJ primers was used. Compared with the method described by Chawade et al. [41], the formula for calculating mutation frequency in this study was modified as that some lines may have more than one mutant band instead of only one mutant band in the same test lines reported by Chawade et al. [41]. The mutation frequency in our population detected by the PCR analysis with RAPD and ISJ primers was estimated at 1/34 kb, which is slightly higher than that estimated by TILLING in the three specific genes, which was 1/47 kb. This difference could reflect the fact that the TILLING method based on specific regions is influenced by more factors, such as chromosomal location, degree of closed or open chromatin, gene redundancy level, GC content, CJE activity, detecting method for digested bands, and the probability of mutations presenting in the effective region (the central 80% of the target sequence) [12], [33]. However, local differences are evened out in the genome wide estimation by PCR with RAPD or ISJ primers and there is no more interference after the PCR amplification. Mutations within the 5′ regions of RAPD or ISJ primers are less likely to affect primer binding [41] and thus the mutation frequency calculated by these methods will be an underestimate. However, the PCR approach with RAPD or ISJ primers is simple, fast and relatively accurate for mutation detection on the DNA level, especially for early estimation.

The utilization of two detection platforms for detecting mutations in TILLING populations was compared here. The most established TILLING techniques adopted fluorescent detection of *CelI* cleavage products on a genetic analyzer (LI-COR genetic analyzer or ABI sequencer), which requires the use of labeled primers in PCR reactions [16], [34]. The non-denaturing polyacrylamide gel system based on silver staining and the agarose system based on ethidium bromide staining, both eliminate the need for expensive genotyping instruments and labeled primers, and are therefore especially compatible with small, low-budget laboratories. Previously, we had developed an agarose gel-based Ecotilling system and verified its effectiveness in screening SNPs in wheat [43]. Other laboratories have also simplified the gel detection system for TILLING. A detailed comparison between agarose gel and LI-COR gel [36], non-denaturing polyacrylamide gel (ethidium bromide staining) and LI-COR gel [12], and the modified agarose TILLING system [2] was reported. Additionally, the silver-staining approach of non-denaturing polyacrylamide system is slightly more sensitive than the ethidium bromide approach and slightly better than the agarose gel system, although the procedure is somewhat slower. However, the non-denaturing polyacrylamide and agarose systems simplify the procedure by only utilizing instrumentation available in any basic molecular biology laboratory and it makes TILLING approach more accessible to a larger set of biologists and crop improvement programs, which may promote the development of multiple wheat TILLING libraries.

To link mutation to phenotype, the online tools SIFT and PARSESNP were used for evaluating the effects of mutation, which predict deleterious effects on an encoded protein and identify changes in restriction endonuclease sites caused by a mutation to facilitate genotypic and phenotypic analysis. Further phenotypic analysis of these mutants could be carried out based on the prediction. The high mutation density in our population (1/47 kb; 16,000 Mb in hexaploid wheat) implies that any given individual is predicted to carry approximately 340,000 mutations. The conventional approach for reducing this large number of undesirable background mutations is to backcross the mutants to the wild type for several generations [2], [11], [12]. These backcross generations are required before the mutations can be used in wheat breeding, because the average performance of the lines generated directly from crosses with the original mutants can be reduced by the background variations [12]. However, the classical backcrossing program is a prolonged procedure, which is cost-extensive in both time and resources, particularly for wheat with long generation time. As an alternative to analyze the changed phenotype, we used sister lines homozygous for the presence or absence of the mutation in a segregating population, in which many of the same background mutations were shared, thus providing a better control than the wild type without background variations [2], [12], [33]. A perfect correlation between the presence of the homozygous mutation and the phenotype would provide strong support for the hypothesis that the novel phenotype is caused by the mutation in the target gene.

In this study, the mutant and non-mutant derivatives from the same M_2_ line were chosen to investigate the phenotypic variation caused by the mutation in photoperiod gene *Ppd-D1*. The p283-1 mutant was crossed to the wild type Jinmai47. Spike development, heading date and flowering date were investigated and compared between the homozygous *Ppd-D1* mutants and non-mutation plants of the F_2_ population. We are now further investigating the effects of the *Ppd-D1* gene on spike development, number of fertile florets and grains per spike. The aim was to produce a new line in which the duration of different phases of spike development was fine-tuned without affecting the anthesis date. This involves altering the duration of the reproductive phase of wheat by manipulating the photoperiod sensitivity of the *Ppd-D1* gene, which may be an alternative to changing the fertile floret number in wheat [44]–[46]. However, even if the desired mutations are not obtained in the current screen, there are many additional lines from the TILLING population available for testing. Overall, TILLING is a flexible strategy for manipulating gene functions by producing a large allelic series of mutations which possibly affect protein function, and thereby mutants with partial phenotypic change or intermediate expression of the target gene could be produced, unlike other reverse genetic tools such as T-DNA and RNAi with insertional or silencing knockouts. Clearly, TILLING will be a useful tool for both research in crop improvement and gene function.

There are only a few groups are currently developing TILLING libraries in wheat. These include tetraploid as well as hexaploid populations in several varieties [2], [11], [12], [15]. The development of multiple TILLING resources in wheat will create a more robust, efficient and flexible wheat TILLING platform as targets which are absent in one library could be screened for in other libraries. Thus, more wheat TILLING resources from different ecological regions in different genetic backgrounds are needed for both practical breeding and functional genomics.

Here we have developed a new genetic resource in the wheat ‘Jinmai47’ genetic background by means of EMS mutagenesis. The high mutation density in this hexaploid species provides a particularly favorable platform for both basic science and crop improvement. With the complete wheat genome sequence information likely to become available in the near future, this resource will be especially appealing to researchers at which point time-consuming gene isolation and design of locus-specific primers would become a relatively simple informatics exercise. Our TILLING resource is open to the scientific community and can be accessed for research through contacting the corresponding author. The modified low-cost detection methods with good sensitivity and public access to those available TILLING populations generated in different genetic backgrounds will hopefully make this technology more accessible.

## Materials and Methods

### Mutagenesis and plant growth conditions

A Chinese common wheat cultivar Jinmai47, with higher drought-tolerance and good agronomic traits and widely used in the rainfed winter wheat regions of China, was used to generate the mutant populations. Approximately 3000 seeds were soaked in 0.8% (v/v) EMS solution at the ratio ∼350 grains/100 mL with gentle agitation overnight (∼18 h) at room temperature. After EMS treatment, seeds were thoroughly washed with tap water for 3 hours and then placed at 4°C for 5 days before being transferred to room temperature. The EMS-treated seeds (M_1_) were sown in pots with soil in a greenhouse until harvest. M_2_ seeds were harvested from 1350 M_1_ individuals, threshed and packed separately [2], [12], [15]. Fifteen M_2_ seeds per line were sowed in one meter rows in the field, three individuals per line were harvested from the M_2_ lines, the M_3_ seeds from each M_2_ individual were collected and labeled accordingly. A proportion of M_5_ seeds and some backcross seeds were further screened for phenotypes.

Forward genetic mutant screening was performed on M_2_ and M_3_ families. All visible mutant phenotypes were carefully inspected and recorded in reference to the parent cultivar ‘Jinmai47’ during the plant life cycle. The frequency of the distinguishable phenotypes was recorded. The novel phenotypes were also photographed with a digital camera.

### DNA isolation and preparation of DNA pooling

Genomic DNA from young leaves was isolated from 2610 M_2_ plants using the CTAB method [47]. DNA concentrations were measured using a spectrophotometer and normalized to 50 ng/µL. Then DNA samples from 4 M_2_ plants with equivalent amounts were pooled for initial screening.

### PCR with RAPD and ISJ primers

The six RAPD markers for estimating mutation frequency are list in table 2.

Fifteen random and ISJ primers were obtained based on Song and Henry [48], Jaroslaw et al. [49], Naghavi et al. [50] and Sajid et al. [51]. After initial RAPD-PCRs, six pairs showing stable bands were selected for further screening. The final PCR protocol used was as follows: 1 µL 10×Taq Buffer (TaKaRa), 1.0 µL dNTPs (2.5 mM each dNTP), 0.6 µL MgCl_2_ (25 mM), 0.5 µL random primer (10 µM), 1 µL DNA (50 ng/µL), and 0.1 µL Taq DNA polymerase (5 U/µL, TaKaRa), 5.8 µL deionized water. An initial denaturation at 95°C for 2 min was followed by 40 cycles at 94°C for 20 s, 55°C for 30 s, 72°C for 90 s and a final extension of 72°C for 5 min. The PCR products were separated on 2.5% agarose gels and visualized after ethidium bromide staining using Gel Doc XR (BioRad Laboratories, Inc).

The gain or loss of a band indicates that there would be at least one base changed in the particular genomic sequence to which the primer binds. Similarly, if there is one mutated base present in the binding sites, the band number will be changed correspondingly. A modified formula was used for estimating the mutation frequency as followed.

*Mutation frequency = [(primer length×2)×(number of bands routinely obtained in wild type×number of the test lines)]/number of mutant bands in the test lines.*

### Mutation Screening by TILLING

The genomic sequences of wheat photoperiod genes (*Ppd*, one member of the pseudo-response regulator (*PRR*) family) were previously deposited in GenBank [GenBank accession number: DQ885753], [GenBank accession number: DQ885757], [GenBank accession number: DQ885766], and served as the A, B and D genome copy, respectively. The primer Ppd2D of *Ppd-D1* gene was designed based on variation between the three homoeologous genes to achieve 2D genome specific amplification. The web based program CODDLE (Codons Optimized to Discover Deleterious Lesions; http://www.proweb.org/coddle), combined with the Primer3 software was used to define the best amplicon for TILLING screening, aiming for a expected primer Tm of 60–70°C. CODDLE identifies the region(s) of a user-selected gene which have the highest probability of affecting gene function when mutated by ENU or EMS. The primers designed were as follows: Ppd2D-L: ATTTTAAGGCGCAGAGCTCATGGACAA and Ppd2D-R: AGAGAGC AGACGAAATCGGCTTTTGAA, the final target is 1496 bp in length and covers the fifth, sixth and seventh exons of the *Ppd-D1* gene.

The locus specific primers AF1 (CAACATCAAGGTATGCATCATGACTATTG)/AR3 (CGTCCCGCGAGTTCACAAGCT) and BF1 (ACGTACGACGTGCATCATC ACC)/BR3 (TCCCGCGAGTTCACCAGCC) for the wheat *rubisco activase* gene were specific to the A and B genome of wheat, respectively.

The protocol for the TILLING targets produced through the non-fluorescence methods is similar to that used for the LI-COR detection system. The PCR reactions were performed in a 20 µl volume containing 13.4 µL deionized water, 2 µL 10×Ex Taq Buffer (TaKaRa), 1.4 µL dNTPs (2.5 mM each dNTP), 0.5 µL each of 10 µM forward and reverse primer, 2 µL of pooled DNA, and 0.2 µL Ex Taq DNA polymerase (5 U/µL, TaKaRa). For *PRR* mutation screening, touchdown PCR was conducted using a thermal cycler (Bio-Rad, DNA Engine, PTC200) as follows: 95°C for 2 min, followed by 5 cycles of 94°C for 20 s, an annealing step starting at 73°C for 30 s and decreasing by 1°C per cycle, 72°C for 90 s; then 37 cycles of 94°C for 20 s, 68°C for 30 s, 72°C for 90 s; and finally elongation at 72°C for 5 min; a denaturing and re-annealing step is included at the end of the PCR reaction (99°C for 10 min, followed by 70 cycles of 70°C for 20 s decreasing by 0.3°C per cycle) to allow the formation of heteroduplexes if a mutation is present in the pool. For the *rubisco activase* gene, the PCR profile was the same as for the *Ppd-D1* PCR except the annealing temperature was 65°C. After PCR amplification, samples were digested with celery juice extract (CJE) which was prepared according to Till et al. [23]. Due to the variable activity of different celery juice extracts, the optimal amount of CJE for heteroduplex digestion was determined empirically using targets with known mutations [43]. Digestion reactions for this study were performed in a 20 µL reaction volume containing 10 µL of PCR product, 2 µL 10×CJE Buffer (10 mM Hepes, pH 7.5,10 mM MgSO_4_, 10 mM KCL, 0.002%Triton X-100, and 0.2 µg/mL BSA); 1.2 µL CJE preparation, and 6.8 µL ultrapure water. The digestion was then carried out at 45°C for 45 min. Subsequently, 2 µL 10×Loading Buffer was added to stop the reaction. Samples were then detected by agarose gel and polyacrylamide gel respectively.

The digested products were separated on 2.5% agarose gels with 1×TAE buffer at 115 V for 40 min, stained in an ethidium bromide buffer (1 µg/mL) for 15 min, and then visualized by Gel Doc XR (BioRad Laboratories, Inc.). Total time required is 1 h.

Alternatively, samples were separated on a 8% polyacrylamide gel (Acrylamide∶bis ratio of 19∶1) in 1×TBE buffer at 250 V for 1.5 h, then visualized by staining with silver solution. Total time required is 2.5 h. The silver staining procedure used here was slightly modified from Merrill et al. [52] and Brant et al. [53]. Fixation: 10% acetic acid, 15 min; Impregnation: AgNO_3_ (2 g/L), 15 min; Rinse: H_2_O, 30 s; Development: Na_2_OH (15 g/L), 37% HCOH (1.5 ml/L), Na_2_S_2_O_3_•5H_2_O (2 g/L), 2–5 min; Stop: 10% acetic acid, 5 min.

Samples for ethidium bromide staining were separated on an 8% polyacrylamide gel (Acrylamide∶bis ratio of 19∶1) in 1×TBE running buffer at 250 V for 1.5 h. The gel was stained with ethidium bromide (1 µg/mL). Total time required is 2 h.

Gel images were analyzed visually for the presence of digested bands whose combined size was similar to the original amplified fragment using Adobe Photoshop software (Adobe Systems Inc., San Jose, CA). If a pooled sample was identified as having CJE digestion fragments, each of the samples from the pool was tested. The target fragments of the mutants screened were then sequenced to determine the mutation.

### Phenotypic analysis

Sequence analysis was carried out to determine the effects of mutations based on the probability of affecting protein function. The PARSESNP (Project Aligned Related Sequences and Evaluate SNPs; http://www.proweb.org/parsesnp/) and SIFT (Sorting Intolerant From Tolerant; http://blocks.fhcrc.org/sift/SIFT) programs were used to predict the severity of mutation identified.

To verify the mutation effect of mutant photoperiod gene identified by TILLING, and examine the agronomic traits of some mutants, mutant lines were advanced to the M_4_ or M_5_ generation and the progress of spike differentiation was investigated using a dissection microscope; the heading date and flowering time were also recorded. Additionally, some mutants were crossed to the wild type Jinmai47, and the mutant and non-mutant plants derived from the same line were used to study the phenotypic change.

## References

[1] Chua NH, Tingey SV (2006). Plant biotechnology: Looking forward to the next ten years.. Curr Opin Biotechnol.

[2] Dong C, Dalton-Morgan J, Vincent K, Sharp P (2009). A Modified TILLING Method for Wheat Breeding.. Plant Gen.

[3] International Rice Genome Sequencing Project (2005). The map-based sequence of the rice genome.. Nature.

[4] Jaillon O, Aury JM, Noel B, Policriti A, Clepet C (2007). The grapevine genome sequence suggests ancestral hexaploidization in major angiosperm phyla.. Nature.

[5] Eversole K (2009). Advancements towards sequencing the bread wheat genome: An update of the projects of the international wheat genome sequencing consortium.. Annual Wheat Newsletter.

[6] Minoia S, Petrozza A, D'Onofrio O, Piron F, Mosca G (2010). A new mutant genetic resource for tomato crop improvement by TILLING technology.. BMC Research Notes.

[7] Hirochika H (2001). Contribution of the Tos17 retrotransposon to rice functional genomics.. Curr Opin Plant Biol.

[8] Weigel D, Ahn JH, Blázquez MA, Borevitz JO, Christensen SK (2000). Activation tagging in *Arabidopsis*.. Plant Physiol.

[9] Alonso JM, Stepanova AN, Leisse TJ, Kim CJ, Chen H (2003). Genome-wide insertional mutagenesis of *Arabidopsis thaliana*.. Science.

[10] Jeon J, Lee S, Jung KH, Jun SH, Jeong DH (2000). T-DNA insertional mutagenesis for functional genomics in rice.. Plant J.

[11] Parry MAJ, Madgwick PJ, Bayon C, Tearall K, Hernandez-Lopez A (2009). Mutation discovery for crop improvement.. Journal of Experimental Botany.

[12] Uauy C, Paraiso F, Colasuonno P, Tran RK, Tsai H (2009). A modified TILLING approach to detect induced mutations in tetraploid and hexaploid wheat.. BMC plant Biology.

[13] Fu D, Uauy C, Blechl A, Dubcovsky J (2007). RNA interference for wheat functional gene analysis.. Transgenic Res.

[14] Fox JL (2004). Monsanto cuts GM wheat.. Nat Biotechnol.

[15] Slade AJ, Fuerstenberg SI, Loeffler D, Steine MN, Facciotti D (2005). A reverse genetic, nontransgenic approach to wheat crop improvement by TILLING.. Nat Biotechnol.

[16] McCallum CM, Comai L, Greene EA, Henikoff S (2000). Targeting induced local lesions in genomes (TILLING) for plant functional genomics.. Plant physiol.

[17] Henikoff S, Till BJ, Comai L (2004). TILLING, traditional mutagenesis meets functional genomics.. Plant Physiology.

[18] Greene EA, Codomo CA, Taylor NE, Henikoff JG, Till BJ (2003). Spectrum of chemically induced mutations from a large-scale reverse-genetic screen in *Arabidopsis*.. Genetics.

[19] Till BJ, Cooper J, Tai TH, Colowit P, Greene EA (2007). Discovery of chemically induced mutations in rice by TILLING.. BMC Plant Biol.

[20] Till BJ, Comai L, Henikoff S, Varshney RK, Tuberosa R (2007). Tilling and EcoTILLING for crop improvement.. Genomics-Assisted Crop Improvement. Genomics Approaches and Platforms.

[21] Sabetta W, Alba V, Blanco A, Montemurro C (2011). SunTILL: a TILLING resource for gene function analysis in sunflower.. Plant Methods.

[22] Rothe N (2010). Validation of tilling populations in diploid and hexaploid wheat. M.S. thesis.

[23] Till BJ, Reynolds SH, Weil C, Springer N, Burtner C (2004). Discovery of induced point mutations in maize genes by TILLING.. BMC Plant Biol.

[24] Caldwell DG, McCallum N, Shaw P, Muehlbauer GJ, Marshall DF (2004). A structured mutant population for forward and reverse genetics in Barley (*Hordeum vulgare L.*).. Plant J.

[25] Talamè V, Bovina R, Sanguineti MC, Tuberosa R, Lundqvist U (2008). TILLMore, a resource for the discovery of chemically induced mutants in barley.. Plant Biotechnol J.

[26] Gottwald S, Bauer P, Komatsuda T, Lundqvist U, Stein N (2009). TILLING in the two-rowed barley cultivar ‘Barke’ reveals preferred sites of functional diversity in the gene HvHox1.. BMC Research Notes.

[27] Wu JL, Wu C, Lei C, Baraoidan M, Bordeos A (2005). Chemical-and irradiation-induced mutants of indica rice IR64 for forward and reverse genetics.. Plant Mol Biol.

[28] Xin Z, Wang ML, Barkley NA, Burow G, Franks C (2008). Applying genotyping (TILLING) and phenotyping analyses to elucidate gene function in a chemically induced sorghum mutant population.. BMC Plant Biology.

[29] Cooper JL, Till BJ, Laport RG, Darlow MC, Kleffner JM (2008). TILLING to detect induced mutations in soybean.. BMC Plant Biol.

[30] Muth J, Hartje S, Twyman RM, Hofferbert HR, Tacke E (2008). Precision breeding for novel starch variants in potato.. Plant Biotechnology J.

[31] Knoll JE, Ramos ML, Zeng Y, Holbrook CC, Chow M (2011). TILLING for allergen reduction and improvement of quality traits in peanut (*Arachis hypogaea L.*).. BMC Plant Biology.

[32] Wang N, Wang Y, Tian F, King GJ, Zhang C (2008). A functional genomics resource for *Brassica napus*: development of an EMS mutagenized population and discovery of FAE1 point mutations by TILLING.. New Phytol.

[33] Stephenson P, Baker D, Girin T, Perez A, Amoah S (2010). A rich TILLING resource for studying gene function in *Brassica rapa*.. BMC Plant Biology.

[34] Till BJ, Colbert T, Tompa R, Enns LC, Codomo CA (2003). High-throughput TILLING for functional genomies.. Methords Mol Biol.

[35] Suzuki T, Eiguchi M, Kumamaru T, Satoh H, Matsusaka H (2008). MNU-induced mutant pools and high performance TILLING enable finding of any gene mutation in rice.. Mol Genet Genomics.

[36] Raghavan C, Naredo MEB, Wang HH, Atienza G, Liu B (2007). Rapid method for detecting SNPs on agarose gels and its application in candidate gene mapping.. Mol Breeding.

[37] Oleykowski CA, Mullins CRB, Godwin AK, Yeung AT (1998). Mutation detection using a novel plant endonuclease.. Nucleic Acids Research.

[38] Bentley A, MacLennan B, Calvo J, Dearolf CR (2000). Targeted recovery of mutations in Drosophila.. Genetics.

[39] Piron F, Nicolaï M, Minoïa S, Piednoir E, Moretti A (2010). An Induced Mutation in Tomato eIF4E Leads to Immunity to Two Potyviruses.. PLoS ONE.

[40] Dahmani-Mardas F, Troadec C, Boualem A, Lévêque S, Alsadon AA (2010). Engineering Melon Plants with Improved Fruit Shelf Life Using the TILLING Approach.. PLoS ONE.

[41] Chawade A, Sikora P, Bräutigam M, Larsson M, Vivekanand V (2010). Development and characterization of an oat TILLING-population and identification of mutations in lignin and β-glucan biosynthesis genes.. BMC Plant Biology.

[42] Nomura T, Ishihara A, Yanagita RC, Endo TR, Iwamura H (2005). Three genomes differentially contribute to the biosynthesis of benzoxazinones in hexaploid wheat.. Proc Natl Acad Sci USA.

[43] Chen L, Wang SQ, Hu YG (2011). Detection of SNPs in the VRN-A1 gene of common wheat (*Triticum aestivum L.*) by a modified Ecotilling method using agarose gel electrophoresis.. Australian Journal of Crop Science.

[44] Halloran GM, Pennell AL (1982). Duration and rate of development phases in wheat in two environments.. Ann Bot.

[45] Slafer GA, Rawson HM (1996). Responses to photoperiod change with phenophase and temperature during wheat development.. Field Crops Res.

[46] González FG, Slafer GA, Miralles DJ (2005). Pre-anthesis development and number of fertile florets in wheat as affected by photoperiod sensitivity genes Ppd-D1 and Ppd-B1.. Euphytica.

[47] Aldrich C (1993). CTAB DNA extraction from plant tissues.. Plant Mol Biol Rep.

[48] Song W, Henry RJ (1995). Molecular analysis of the DNA polymorphism of wild barley (*Hordeum spontaneum*) germplasm using the polymerase chain reaction.. Genetic Resources and Crop Evolution.

[49] Przetakiewicz J, Nadolaka-orczy A, Orczyk W (2002). The use of RAPD and semi-random markers to verify somatic hybrids between diploid lines of *Solanum tuberosum L.*. Cellular & Molecular Biology Letters.

[50] Naghavi MR, Mardi M, Ramshini HA, Fazelinasab B (2004). Comparative analyses of the genetic diversity among bread wheat genotypes based on RAPD and SSR markers.. Iranian Journal of Biotechnology.

[51] Bibi S, Dahot MU, Khan IA, Khatri A, Naqvi MH (2009). Study of genetic diversity in wheat (*Triticum Aestivum L.*) using random amplified polymorphic DNA (RAPD) markers.. Pak J Bot.

[52] Merril CR, Harrington M, Alley V (1984). A photodevelopment silver stain for the rapid visualization of proteins separated on polyacrylamide gels.. Electrophoresis.

[53] Bassam BJ, Caetano-Anollés G, Gresshoff PM (1991). Fast and sensitive silver staining of DNA in polyacrylamide gels.. Analytical Biochemistry.

